# Effects of washing agents on the mechanical and biocompatibility properties of water-washable 3D printing crown and bridge resin

**DOI:** 10.1038/s41598-024-60450-7

**Published:** 2024-04-30

**Authors:** Yunqi Liu, Gan Jin, Jung-Hwa Lim, Jong-Eun Kim

**Affiliations:** Department of Prosthodontics, College of Dentistry, Yonsei University, Yonsei-ro 50-1, Seodaemun-gu, Seoul, 03722 Republic of Korea

**Keywords:** Dentistry, Dental materials, Prosthetic dentistry, Restorative dentistry

## Abstract

Three-dimensional (3D) printing, otherwise known as additive manufacturing in a non-technical context, is becoming increasingly popular in the field of dentistry. As an essential step in the 3D printing process, postwashing with organic solvents can damage the printed resin polymer and possibly pose a risk to human health. The development of water-washable dental resins means that water can be used as a washing agent. However, the effects of washing agents and washing times on the mechanical and biocompatibility properties of water-washable resins remain unclear. This study investigated the impact of different washing agents (water, detergent, and alcohol) and washing time points (5, 10, 20, and 30 min) on the flexural strength, Vickers hardness, surface characterization, degree of conversion, biocompatibility, and monomer elution of 3D printed samples. Using water for long-term washing better preserved the mechanical properties, caused a smooth surface, and improved the degree of conversion, with 20 min of washing with water achieving the same biological performance as organic solvents. Water is an applicable agent option for washing the 3D printing water-washable temporary crown and bridge resin in the postwashing process. This advancement facilitates the development of other water-washable intraoral resins and the optimization of clinical standard washing guidelines.

## Introduction

The worldwide digital revolution and associated developments in manufacturing have resulted in rapid advancements in three-dimensional (3D) manufacturing techniques in recent years^1^. Additive manufacturing (AM) is defined as “a method based on adding materials layer upon layer to form 3D objects from 3D model data” by the American Society for Testing and Materials^2^. AM has found significant use in the medical field due to its capacity to control the addition of materials and customize complex geometries that cater to the specific requirements of individual patients with different conditions^3,4^. Some of the most common medical applications of AM include producing medical models, implants, and surgical instruments^4^. Specifically, AM can obtain patient geometry through data acquisition methods such as CT scans, and rebuild real anatomical models. Not only does it provide surgeons with preoperative practice opportunities, but it can also be used to design patient-specific implants^3^. However, AM processing in medical applications can cause damage to mechanical properties, resulting in undesirable transformations and distortion^4^. In dentistry, additive manufacturing offers a variety of material options like polymers, ceramics, and alloys, and can be used to produce splints, orthodontic appliances, and surgical guides^5^.

3D printing, using standardized materials to create individual 3D objects through a specific automated process, supported by a computer-aided design digital model, is basically an AM process^6^. Vat polymerization is a popular 3D printing additive manufacturing technology in the dental field^7^. Two representative methods are stereolithography apparatus (SLA) and digital light processing (DLP), with a slight difference. The SLA printer performs ultraviolet (UV) laser to polymerize photocurable resin and manufacture high-accuracy products^8^, but the process lacks time efficiency as the object is created one layer at a time^7^. The DLP printer uses high-resolution digital light projectors to harden the liquid resin with plane-exposure molding technology, which makes it possible to cure an entire layer each time the light beam is transmitted and thereby speed up the production process^7,9^. This has the advantages of fast processing, low cost, and high resolution^10^. In dentistry, DLP printers can create objects such as restorations, implants, aligners, models, surgical guides, and splints^9,11^.

The workflow of producing dental restorations with a 3D printer using photopolymerizable resin can be summarized into printing, postwashing, and postcuring^12^. After finishing the printing process, the object must undergo postwashing to remove any residual uncured resin that may adhere to its surface^13^. An effective washing method is to rinse or immerse the printed object in an organic solvent, which is usually isopropyl alcohol (IPA), tripropylene glycol monomethyl ether (TPM), or ethyl alcohol (ethanol)^14^. This is because the main oligomers or monomers in most resin components are only soluble in organic solvents, allowing the adherent residual resin to be quickly and strongly washed away^15^. However, the use of organic solvents has disadvantages. The previous study observed that the mechanical properties of the 3D printed objects can be weakened by the alcohol-based solvent after the extended washing process^16^. Also, the high volatility of alcohol leads to its accumulation in the air, causing high indoor concentrations and impairing human health^17^. These issues associated with organic-solvent-based washing have resulted in the 3D printing industry exploring and developing organic-solvent-free postwashing methods.

Novel water-washable 3D printing resins make it possible to use water to wash the printed objects^18^. The principle involves constructing a waterborne, water-soluble, and hydrophilic photosensitive curable resin^19–21^ to allow the residual unpolymerized resin on the surface of the printed object to be dissolved in water and washed away^20^ before performing UV-light postcuring^15^. Waterborne resin raw materials and formulations can help achieve these goals. One current common approach is to use waterborne polyurethane and waterborne polyurethane acrylate as the main raw materials to make water-washable 3D printing photosensitive resin, and achieve the hydrophilic effect by adding hydrophilic groups: –OH, –COOH, and –NH^+^^19^. The advantages of using water-washable resin include reducing the amount of volatile organic compounds involved, benefiting human health and the working environment^15,22^, and improving cost-effectiveness by not needing the manufacturer’s chemical reagent.

Water-washable 3D printing resin materials have also been introduced into the dental field. Dental water-washable 3D printing model resins and temporary crown and bridge (C&B) resin materials are now commercially available. Water-washable temporary C&B resins need more attention, since dental-model resin materials only need to be used extraorally, whereas a temporary C&B resin is an intraoral restorative material requiring mechanical properties consistent with the applied occlusal forces and also biocompatibility^23^. Previous studies have shown that increasing the postwashing time with commonly used organic-based solvents improves the cytocompatibility of printed objects^16,24^, but this also loosens the polymer network structure and hence degrades the mechanical properties^25^. In contrast, polymethyl methacrylate (PMMA) was found to not exhibit relaxation behavior and plastic deformation after long-term immersion in water^26^. Therefore, extended water washing of the printed object might preserve its original mechanical properties and ensure biocompatibility.

To the best of the authors’ knowledge, little research has been done in this area of dentistry. Further studies are therefore required to evaluate the washing ability of water in removing residual monomer on the surface of objects produced using water-washable resin, and comparing this with using a conventional organic-based solvent. There is also a need to determine the combined influence of water agents and washing time on both the mechanical and biocompatibility properties of water-washable resin printed objects.

This study investigated the influence of different washing agents on a water-washable 3D printing C&B resin under different washing times. The null hypothesis was that the use of different washing agents (95% ethanol, water, and 2% detergent) and different washing times has no effect on the mechanical properties and biocompatibility of water-washable 3D printing C&B resins.

## Results

### Mechanical tests

**Figure 1:**
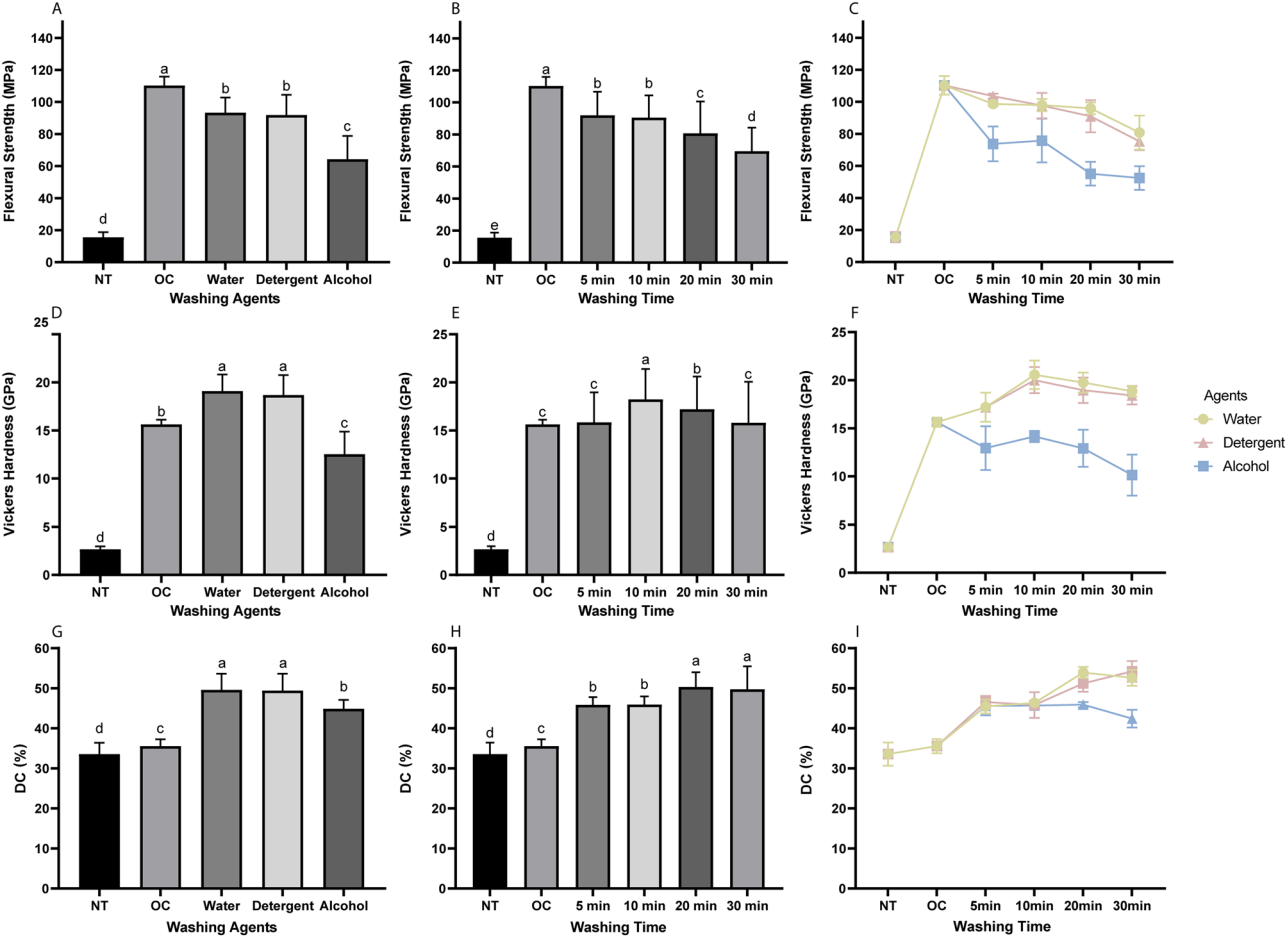
Results of mechanical tests and DC: (**A, B**) Results from a two-way ANOVA of the flexural strength according to washing agents and washing times. (**C**) Trends of flexural strength for different washing times and washing agents in water-washable C&B resin material. (**D, E**) Results from a two-way ANOVA of Vickers hardness according to washing agents and washing times. (**F**) Trends of Vickers hardness of water-washable C&B resin material for different washing times and washing agetns. (**G, H**) Results from a two-way ANOVA of the DC according to washing agents and washing times. (**I**) Trends of the DC of water-washable C&B resin material for different washing times and washing agents. Data are mean and standard-deviation values. Different lower-case letters indicate significant differences.

A two-way ANOVA was used to evaluate the effects of washing agents and washing times on flexural strength. The flexural strength was significantly affected by the washing agents (*F* = 2490.58, *p* < 0.001) and washing times (*F* = 1244.53, *p* < 0.001), with a significant interaction between these parameters (*F* = 52.64, *p* < 0.001) (Fig. 1A,B).

The flexural strength was highest in the OC group (110.31 ± 5.55 MPa), followed by the water and detergent groups, and lowest in the alcohol group (64.30 ± 14.50 MPa) (Fig. 1A). The flexural strength gradually decreased as the washing time increased, and was lowest in the 30-min washing group (69.53 ± 14.75 MPa) (Fig. 1B).

Gradual decreases in the flexural strength with increasing washing time in different solutions are evident in Fig. 1C. The flexural strength in the detergent group decreased steadily with increasing washing time, and was superior to that in the water group before 10 min and lower than that in the water group after 10 min. The flexural strength in the water group had significantly decreased at 30 min and had remained stable before that. The flexural strength in the alcohol group was more severely reduced by the postwashing process, and was significantly lower than that in the other two groups at every time point, and significantly decreased at 20 min.

A two-way ANOVA was used to evaluate the effects of washing agents and washing times on the Vickers hardness. The Vickers hardness was significantly affected by the washing agents (*F* = 2490.58, *p* < 0.001) and washing times (*F* = 1244.531, *p* < 0.001), with a significant interaction between these parameters (*F* = 52.642, *p* < 0.001) (Fig. 1D,E).

The Vickers hardness was highest in the water (19.09 ± 1.73 GPa) and detergent (18.69 ± 2.07 GPa) groups, followed by the OC group (15.63 ± 0.48 GPa) and then the alcohol group (12.53 ± 2.35 GPa) (Fig. 1D). The Vickers hardness peaked at 10 min (18.24 ± 3.16 GPa) and then gradually decreased, with the values after 5 min and 30 min not differing significantly from that in the OC group (Fig. 1E). The Vickers hardness was lowest in the NT group (2.66 ± 3.12 GPa).

The trends of the Vickers hardness for different washing times in different washing solutions are shown in Fig. 1F. Washing increased the Vickers hardness in both the water and detergent groups, resulting in it being higher in the washed samples than in the OC group (15.63 ± 0.48 GPa). The Vickers hardness initially increased with the washing time in these two groups, peaking at 10 min, and then decreased slowly thereafter. In contrast, washing with alcohol reduced the Vickers hardness, with this being lower in all washed groups than in the OC group. The Vickers hardness in the alcohol group peaked at 14.15 ± 0.58 GPa after 10 min of washing, and then decreased gradually to a lowest value of 10.14 ± 2.12 GPa after 30 min. The average Vickers hardness values in the water and detergent groups were superior to those in the alcohol group for all washing times.

### Degree of conversion

A two-way ANOVA indicated that the DC differed significantly with washing agents (*F* = 915.8, *p* < 0.01) and washing times (*F* = 631.92, *p* < 0.01), with a significant interaction between these parameters (*F* = 31.79, *p* < 0.01) (Fig. 1G,H).

The DC was significantly higher in both the water (49.58 ± 4.06%) and detergent (49.44 ± 4.20%) groups than in the alcohol group (44.89 ± 2.21%) (Fig. 1G). The DC gradually increased with the washing time, to reach 50.33 ± 3.68% after 20 min and remaining stable thereafter (Fig. 1H).

The DC showed the same stable trend when washing in different agents before 10 min and then differentiated thereafter (Fig. 1I). The water and detergent groups showed upward trends after 10 min, peaking at 53.94 ± 1.40% after 20 min and at 54.20 ± 2.59% after 30 min, respectively. Conversely, the DC decreased to 42.42 ± 2.21% after 30 min of washing in the alcohol group.

### Scanning electron microscopy

**Figure 2:**
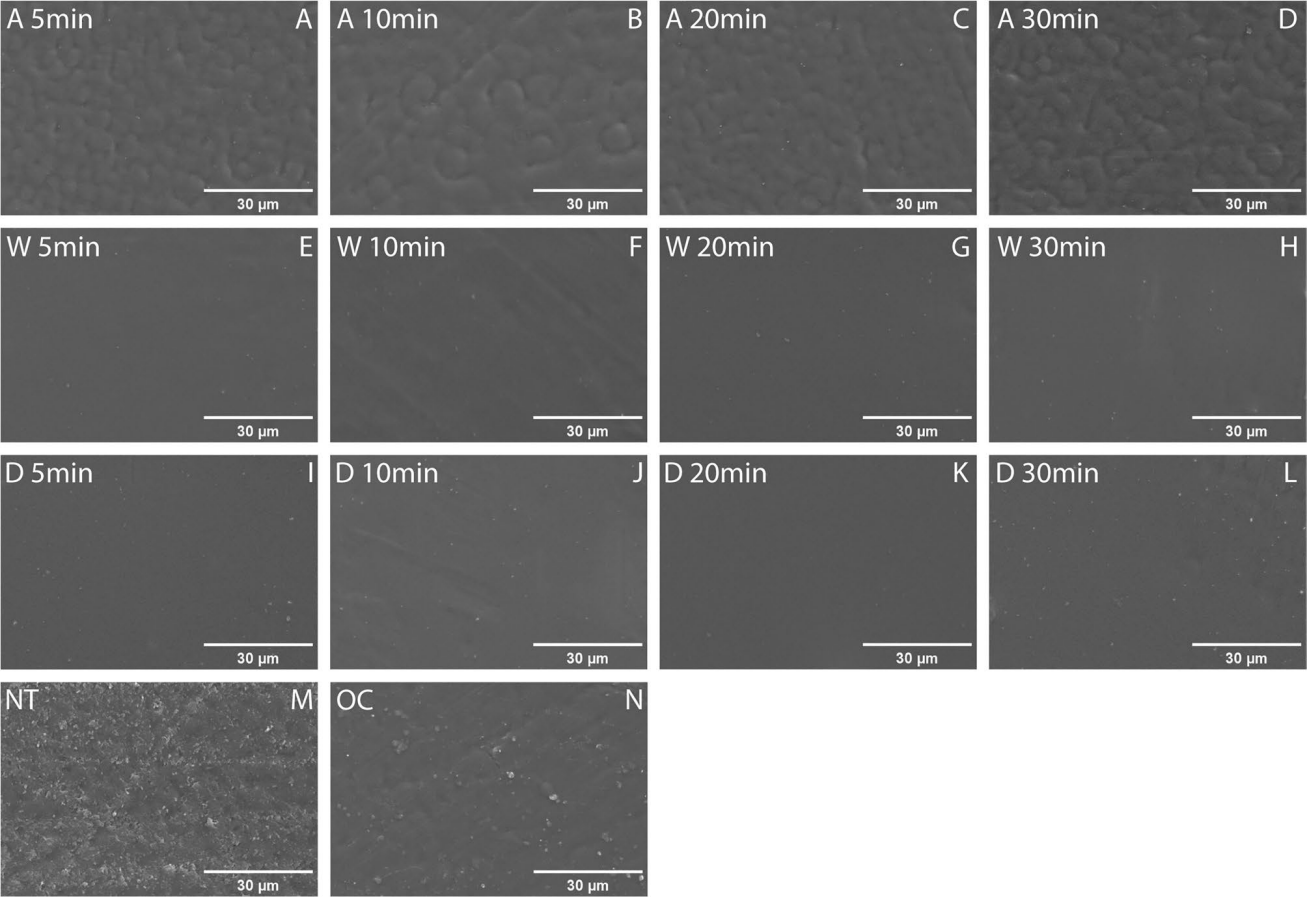
SEM micrographs: SEM micrographs of samples with different washing agents and time. (Magnification 1500× ; scale bar = 30 µm) *A*, alcohol (**A–D**); *W*, water (**E–H**); *D*, detergent (**I–L**); *NT* non-treatment (**M**); *OC* only-cured (**N**).

Figure 2 presents a comparison of the surface characterization of all specimens. The samples from the alcohol group had the roughest surface compared to other washing agents (Fig. 2A–D). The samples from the group washed with water and detergent group showed similar smooth surfaces (Fig. 2E–L). The NT group exhibited the most obvious texture (Fig. 2M).

### Biocompatibility test: cell viability

**Figure 3:**
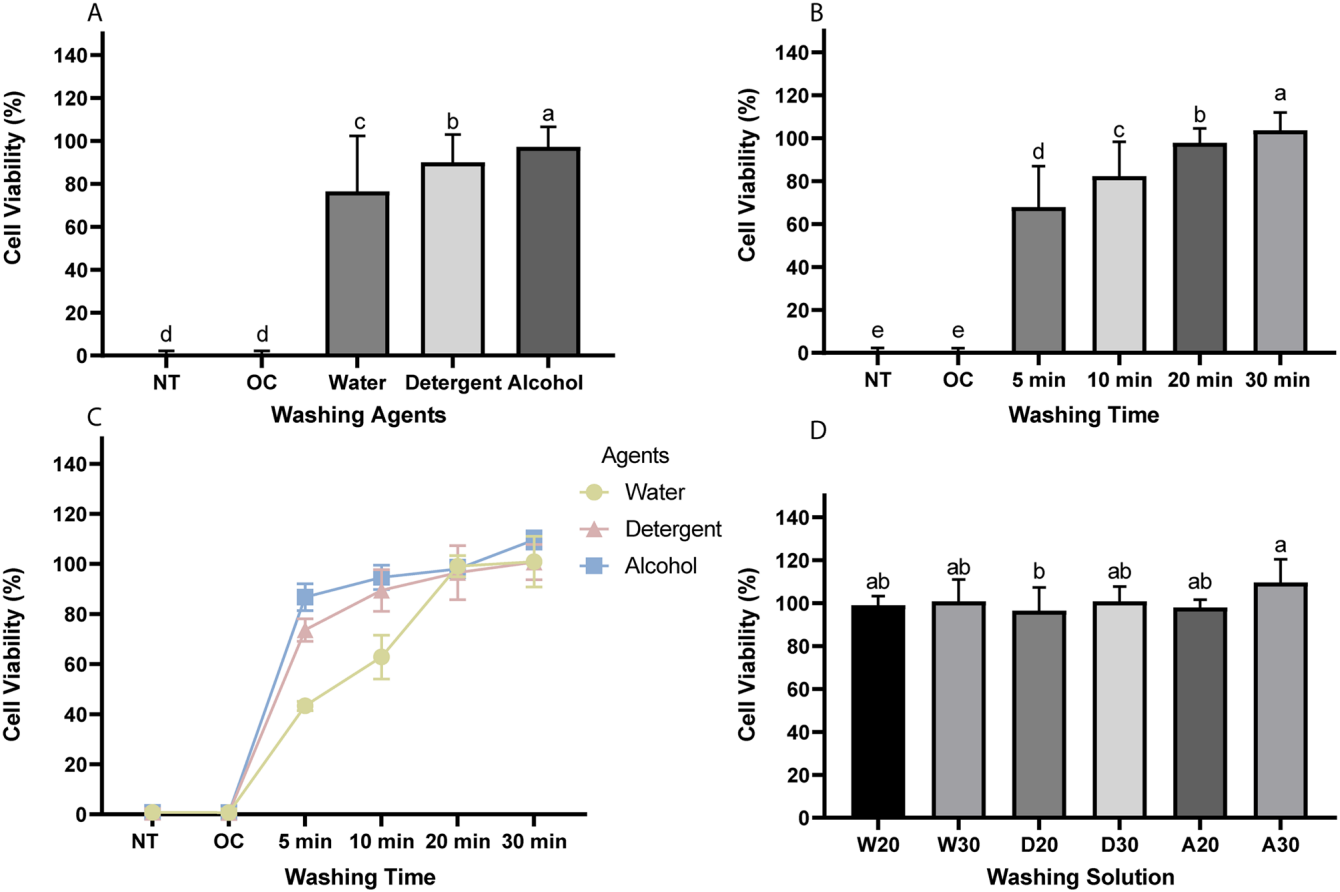
Results of cell viability: (**A, B**) Results from a two-way ANOVA of the cell viability according to the washing agents and washing times. (**C**) Trends of cell viability for different washing times and washing agents applied to water-washable C&B resin material. (**D**) Results from a one-way ANOVA of cell viability of the six separate groups washed in water (W), detergent (D), and alcohol (A) for 20 min (20) and 30 min (30). Data are mean and standard-deviation values. Different lower-case letters indicate significant differences.

A two-way ANOVA was used to evaluate the effects of washing agents and washing times on cell viability. The cell viability was significantly affected by the washing agents (*F* = 2362.34, *p* < 0.001) and washing times (*F* = 1503.675, *p* < 0.001), with a significant interaction between these parameters (*F* = 35.415, *p* < 0.001) (Fig. 3A,B).

The cell viability was highest in the alcohol group (97.23 ± 9.35%), followed by the detergent group (90.05 ± 12.93%) and then the water group (76.52 ± 25.79%) (Fig. 3A). All of the cells in the NT and OC groups died, with measured viability values of 0.76 ± 1.46% and 0.65 ± 1.47%, respectively. With the exception of the groups in which all cells died, the cell viability was lowest after 5 min of washing (67.88 ± 19.05%), and then gradually increased with the washing time, peaking at 103.72 ± 8.23% after 30 min (Fig. 3B).

The cell viability increased for all agents with the washing time (Fig. 3C). Before washing for 20 min there were significant discrepancies in cell viability among the three agent groups, being higher in the alcohol group than in the detergent group, and lowest in the water group. However, the water group exhibited the largest increase in cell viability over time. After 20 min, the cell viability in the water group (99.08 ± 4.22%) was slightly higher than that in the alcohol group (97.95 ± 3.57%) and the detergent group (96.50 ± 10.79%), although without a significant difference.

Figure 3C shows that the mean values of cell viability for the different agents were very close after 20 min and 30 min. Therefore, a one-way ANOVA of the specific washing agents and washing times as an independent group of washing solutions was carried out (Fig. 3D). Figure 3D shows that the cell viability peaked at 109.59 ± 3.61% in the 30 min alcohol group, with a significant difference only relative to the 20 min detergent group.

### Cell morphology analysis

**Figure 4:**
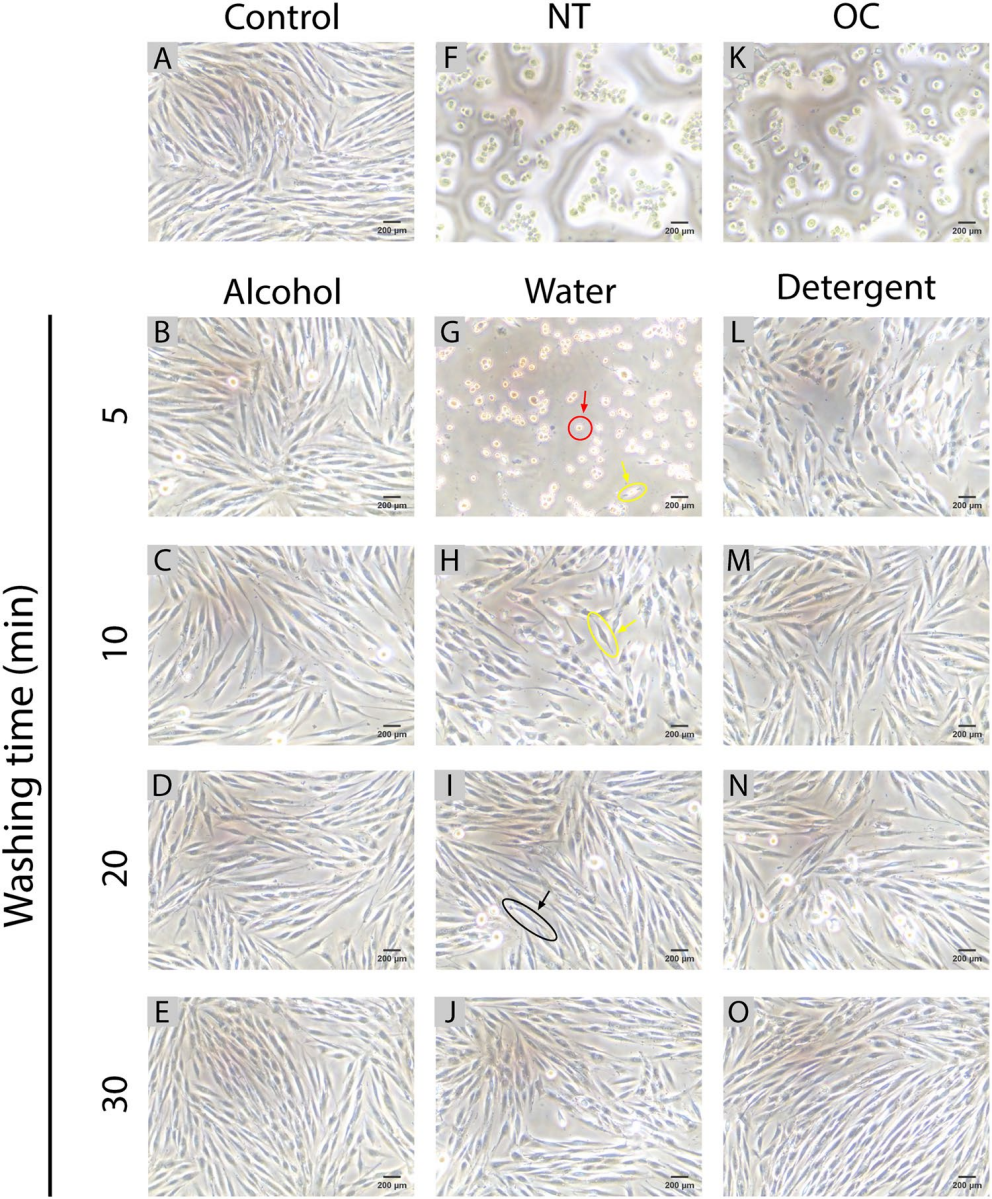
Cell morphology: Inverted microscopy images of HGFs cultured at (8 × 10^4^ cells/mL) in the sample extracts for different washing agents and washing times after 24 h of incubation (scale bar = 200 µm). (**A**) control group, (**F**) non-treatment group, (**K**) only-cured group, (**B–E**) alcohol groups, (**G–J**) water groups, (**L–O**) detergent groups. Notable features are annotated for (**G–J**): round-shaped cell (marked by red circle and arrow), short-spindle shaped cell (highlighted with yellow circles and arrows), elongated-spindle shaped cell (marked by black circle and arrow).

The morphology and density of cells cultured in the specimens’ extracts were observed using an inverted microscope (Fig. 4).

The control group served as a positive control group and represented the cells grown in DMEM for 48 h without changing any resin extracts (Fig. 4A). The cells were shaped like elongated spindles, highly adherent, and in close contact with adjacent cells. The cells in the alcohol-washed groups were similar to those in the control group, as seen in Fig. 4B–E. The GS and OC groups served as the negative control groups, all cells in these groups died (Fig. 4F,K).

In the water groups, the main discrepancy was reflected in the morphologies, sizes, and densities of HGFs as the washing time increased. In the 5 min water-washed group, the cells were round and scattered, with occasional short spindle-shaped cells intermingled (Fig. 4G). After water washing for 10 min, most of the fibroblasts appeared to have a short spindle shape and their contacts with neighboring cells became closer (Fig. 4H). After washing for 20 min and 30 min, the density and number of cells both increased (Fig. 4I,J). The shape of the fibroblasts became like elongated spindles, as observed in the control group.

### Gas chromatography analysis

Table 1 presents the GC/MS detection results. The specific concentrations of all groups, except for NT, were below the detection limit. TEGDMA was detected in the NT group at a concentration of 135 ppm.

**Table 1:** The residual monomers eluted in acetone after 24 h were quantified.

Compound	HEMA			MMA			TEGDMA		
Agent	W	D	A	W	D	A	W	D	A
NT	11			835			346,304 (135 ppm)		
OC	8			813			82		
Time									
5 min	16	11	17	765	752	325	282	321	1752
10 min	8	–	8	873	775	598	436	196	737
20 min	5	9	7	808	889	601	404	128	377
30 min	5	14	2	890	670	746	590	207	230

Peak area/Counts, *n* = 3.

The value of the integral peak area was used to represent the amount of released quantities.

“–” indicates that the corresponding area was below the detection limit of the device.

*W* water, *D* detergent; *A* alcohol.

## Discussion

This study focused on evaluating the effects of washing agents and washing times on the mechanical properties and biocompatibility of a 3D printing water-washable temporary C&B resin. We found that the type of washing agent and washing time both significantly influenced the biological and mechanical properties of 3D printed samples, and so the null hypothesis was rejected.

The evaluations of mechanical properties revealed that flexural strength and Vickers hardness were higher in the water and detergent groups than in the alcohol group at every time point. Alcohol had a more obvious negative impact on the mechanical properties of 3D dental resin products during the washing process, and this impact increased with time, and especially affected the flexural strength. Due to its diffusion in the PMMA material and component leaching, alcohol creates voids in the material^27^ that allow additional solvent to be absorbed and diffused. This lowers the glass transition temperature, leading to phenomena such as plasticization and crazing of the PMMA polymer^28^. A previous study showed that contact with free alcohol causes softening of PMMA and mechanical deformations such as the swelling of sample edges^26^. The mechanical properties would be weakened by the solvent molecules, reducing polymer entanglements and destroying interchain bonds, which cause degradation of the matrix–filler interface^29,30^. Conversely, the present study found that alcohol-free agents could better preserve the original mechanical properties or even enhance the Vickers hardness. A previous study showed that even when the polymer was immersed in water for a long period of up for 30 days, although water continued to be absorbed throughout the immersion period, neither relaxation behavior nor plastic deformation could be detected^26^. Although both alcohol and water are known to be absorbed by polymer networks, alcohols would weaken the mechanical properties of the contacted polymer and act more aggressively^31^, potentially even causing visible cracks on the polymer surface^26^. This may explain why the mechanical properties of the samples washed with water were more stable and superior than those washed in alcohol.

SEM evaluation revealed that resin samples washed with alcohol had rougher surfaces compared to the smooth surfaces observed in the water and detergent groups. The common surface characteristics of resins treated with alcohol can be investigated in previous research^32^. It could also be found that PMMA resin experiences greater roughness and lower hardness when rinsed to absolute ethanol compared to distilled water^32^. This is consistent with the results of our experiment that the mechanical properties of the resin in alcohol groups with a rough surface were lower than those of the water groups with a smooth surface. Surface roughness is an important attribute of dental restoration materials that can impact the colonization of microorganisms and plaque adhesion. Highly rough resin surfaces lead to the potential risk of gingival inflammation, secondary caries, and prosthesis discoloration^33^. Under this situation, water-washed resin seems to have a better possibility to resist microbial adhesion, resulting in a superior general clinical restoration effect. However, the related surface roughness and bacterial adhesion are necessary to be assessed in further investigation.

DC represents the degree of monomer conversion to resin polymer, as determined by the ratio of the carbon double bonds (C=C) converted into carbon single bonds (C–C)^34^. Its significance is that a higher DC normally corresponds to a lower level of residual monomers and better biocompatibility^16,35^. We found that DC was higher in all of the washing groups than in the nonwashing group. There was no difference in the value up to 10 min of washing, but it increased after 20 min of washing with water and detergent, and it decreased after 30 min of washing with alcohol. DC increased with the washing time in the water and detergent groups, which is consistent with the results of other studies, and can be attributed to the removal of uncured monomers by washing^16,24,25^. However, alcohol has a stronger swelling capacity and diffuses more easily than water, resulting in the cross-linking density within the polymer decreasing and a lower DC^36,37^. Excessive washing may aggravate these effects^35^, as reflected in the present finding of a significant decrease in DC after 30 min of washing in alcohol. A higher DC is often correlated with a better biological outcome. In the present study, the DC of the resin sample increased significantly after washing, and accordingly the cell viability also improved. However, some studies have produced the contrasting result that DC increased after prolonged immersion in alcohol (6 months) relative to short-term immersion (24 h)^38^. This might be attributable to the solvent expanding the material’s matrix and enhancing mobility, with this in turn facilitating the diffusion of small molecules and free radicals within the matrix to react with the remaining double bonds in the network^39,40^.

The cell viability test results and cell morphology images indicated that all the cells died in the unwashed groups. With a longer washing time the viability of cells increased, and their morphology and density approached those in the control group (Figs. 3C and 4). Cell viability and morphology varied between washing agents up to 20 min of washing time, with alcohol being the most effective agent for eliminating the toxic resin monomer, and water being the least effective (Fig. 3C). However, the effects of washing the resin for 20 min were similar for the different washing agents in terms of cell viability, shape, and density (Figs. 3D and 4). The main ingredients of our resin are urethanes, methacrylates, and phosphine oxide. Previous studies have shown that these ingredients can have cytotoxic and genotoxic effects in their uncured state^41^, and may even cause cell death^42^. This was consistent with our finding that all the cells died in the two groups in which the specimens were not washed. Increased the washing time presumably resulted in higher cell viability due to the removal of uncured monomers from the specimen surfaces^24^.

Before washing for 20 min, the cell viability is much better for alcohol than for water. This aligns with previous studies finding that cell viability was higher in alcohol-treated specimens than in water-treated specimens^42^. Immersing in pure alcohol helps to reduce the residual compounds in the acrylic polymers in temporary restorative resin materials^43^. Alcohol can accelerate the adsorption of water by the polymer matrix and promote the diffusion of residual monomers from the polymer^44^, thereby removing the remaining monomers and improving the biocompatibility of the polymer. Alcohol and organic solvents are better than water at removing unreacted components from dental composites. Organic solvents have a superior ability to extract monomers and oligomers^45^. This explains why alcohol was the most effective agent at washing off toxic substances within 20 min in this study, while water is the least effective agent. According to ISO 10993-5, biological material extracts associated with a cell viability exceeding 70% of the control are considered nontoxic^46^. After 20 min of washing, the mean cell viability in all the present groups exceeded 90%, with the water-washing group reaching 99%. Washing specimens with water for a sufficient time results in the waterborne resin monomer on the polymer surface being dissolved and eliminated, making the material noncytotoxic; 20 min was a significant time point for washing off the water-washable C&B resin since both organic solvents and water had equal and sufficient washing effects for longer washing times.

Appropriate methods should be used to detect and quantify eluted monomers that cause cytotoxicity in resin composites^47^. GC–MS is the recommended quantitative analysis method for detecting low-molecular-weight eluting monomers^48^. In accordance with recommendations of Jin et al.^35^, three cytotoxic monomers commonly used in 3D printing resins were evaluated: methyl methacrylate (MMA), 2-hydroxyethyl methacrylate (HEMA), and triethylene glycol dimethacrylate (TEGDMA). The TEGDMA monomer could be detected in the NT group but not in the other groups, making it impossible to quantify. The peak area was used to interpret the results because the content of each monomer is proportional to the area of its peak (Table 1)^49^.

The present C&B resin formula contained 15% HEMA, but its peak areas were close to zero. However, undetectable compounds may still be released, causing cytotoxicity^50^. After washing with water and detergent, there was almost no difference in the amounts of MMA monomer released in the NT and OC groups, and the residual MMA content remained relatively stable over time. This suggested that washing does not effectively remove residual MMA monomer. However, when washed with alcohol, the residual MMA content was relatively low after a short washing time but increased slightly as the washing time increased. MMA is a hydrophobic monomer^51^, and so is more soluble in alcohol. A short washing time with alcohol caused a large amount of monomer on the resin surface to be dissolved and removed, but the alcohol penetrated and swelled the polymer network as time passed, causing the monomer to leach out and thereby increase the residual amount^52^. This is consistent with another study finding that the amount of MMA leached from acrylic resin increased with the immersion time in alcohol, but remained constant when immersed in water^53^. In all of the present washing groups, the residual TEGDMA monomer was significantly reduced compared with the NT group. TEGDMA is a more hydrophilic monomer^54^ than the organic solvent alcohol, making it easier to remove in an aqueous agent such as water, and therefore showing a lower residual level. However, the amount of monomer detected in all of the washing groups in this study was far below the level that causes cytotoxicity.

The postwashing process allows the monomers on the resin surface to be dissolved and removed by the washing agents^13^. But immersion in a solvent can also cause the polymer network to swell and release residual monomers^55^. These two opposing trends may explain the different levels and trends of monomer residues reported above. In addition, hydrophobic monomers are more soluble in organic solvents, while hydrophilic monomers are more attracted to aqueous agents^55^. Monomers dissolve easily in agents with the same characteristics, resulting in lower residual levels within a short washing time. Diffusion is promoted when the washing agent and resin composition have similar solubility parameters. After prolonged washing, the agent penetrates the resin matrix to cause it to swell and release residual monomers^55^. The leaching process of monomers is also affected by other factors such as the monomer-to-polymer conversion rate, the composition and temperature of the immersion agent, and the size and chemical characteristics of the leachable substances^56^, leading to differences and uncertainties. Since the C&B resin is applied directly inside the mouth, which is a complex environment, the release of monomers can be affected by intraoral factors such as contact with water, saliva, and alcohol, immersion time, and temperature changes^16,55^. Monomer release in the mouth may cause allergic reactions and pathogenic diseases that harm human health^57^. The factors affecting monomer release and the unclear release process mean that more in-depth research is needed in the future.

This study had some limitations. First, only a water-washable C&B resin was tested, and so other C&B resins that are washed using traditional organic solvents should be included in future comparison tests. Second, two washing devices with different operating principles were used in combination, namely ultrasonic and rotary washers. Interactions between these instruments might have caused inaccuracies in the variable of washing time. Third, 95% ethanol was used to represent organic solvents, but there are other commonly used postwashing organic solvents including IPA and TPM, and different organic solvents may have different effects.

## Methods and materials

### Specimen designing and printing

**Figure 5:**
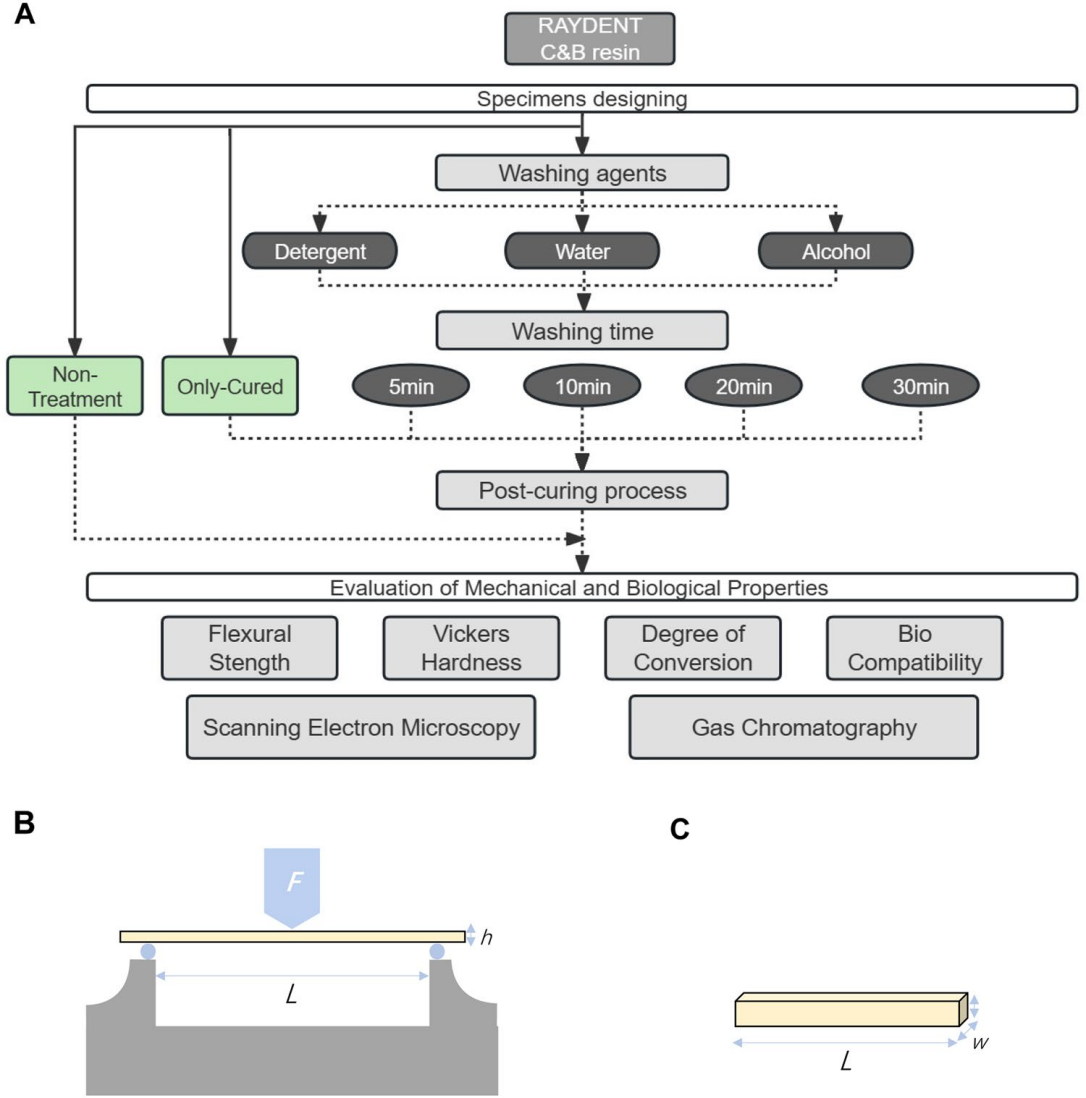
Workflow and schematics: (**A**) Workflow of the study. (**B, C**) Schematics of the test setup for ISO 4049. (**B**) An universal testing instrument. (**C**) The shape of tested specimens.

The workflow of this study is shown in Fig. 5A. A commercially available water-washable 3D printing C&B resin (RayDent, Suwon, Korea) was used (Table 2).

**Table 2:** Material used for 3D printing.

Product	Code	Composition	Manufacturer
RayDent C&B Resin	RD	Urethane acrylate; (octahydro-4,7-methano-1H-indenediyl) bis (methylene) bismethacrylate; HEMA; 2,4,6-trimethylbenzoyl-diphenyl phosphine oxide; 1,6-hexanediol diacrylate; 4-methoxyphenol; color pigments	RayDent, Suwon, Korea

Before printing, specimens of different shapes and sizes were designed for each test. 140 bar-shaped specimens with a length of 25 mm, width of 2 mm, and height of 2 mm were constructed for the flexural strength test (n = 10) and stored in a 37 °C incubator for 24 h according to the ISO 4049 standard. A total of 196 disc-shaped specimens with a diameter of 10 mm and thickness of 2 mm were made for the Vickers hardness test (n = 8) and degree of conversion (n = 6). 84 disc-shaped specimens with a diameter of 15 mm and thickness of 2 mm were designed for biocompatible evaluation (n = 6). For scanning electron microscopy (n = 1), 14 disk-shaped specimens were created with a diameter of 9 mm and thickness of 2 mm. For the gas chromatography (n = 3), 42 specimens applied were disks with a diameter of 6 mm and a thickness of 2 mm.

All specimens were designed using modeling software (Rhino 7, Robert McNeel & Associates, Seattle, WA, USA), and then translated as STL (Standard Tessellation Language) files and printed by a DLP printer (Asiga, Przemek Seweryniak, Cosmodent, Sweden) with a 50-µm layer thickness and no support structure.

### Postwashing and postcuring

Three washing agents were prepared for use in the washing process: tap water, 95% ethanol, and 2% detergent (Table 3). The specimens were divided into four groups and underwent the following two-step washing processes: (1) washing in a rotary washer (Twin tornado, Medifive, Incheon, Korea) respectively in washing agents of tap water, 95% ethanol, and 2% detergent dilution for 3 min, 5 min, 10 min, and 15 min; and (2) immersion in a tank with the corresponding agents followed by washing in an ultrasonic bath (UCP-02, JEIOTECH, Kyungido, Korea) for 2 min, 5 min, 10 min, and 15 min. Specimens in the water and detergent groups were all immersed in water during the second stage of washing. The nontreated (NT) group was set as a control group without postwashing and postcuring. The specimens in the only postcuring (OC) group were post-cured but not washed.

**Table 3:** Washing agents used for postwashing.

Product	Name	Composition	Manufacturer
Tap water	Water	100% tap water	
95% ethanol	Alcohol	CH_3_CH_2_OH	REAGENTS DUKSAN, Ansan, Korea
0.2% dishwashing detergent	Detergent	12% surfactant equivalent: plant-based fatty alcohol (anion), plant-based higher fatty amine (nonionic), fermented native grass	LIONKOREA, Incheon, Korea

After washing, postcuring was performed using a UV-light postcuring device (LC-3DPrint Box, NextDent, Soesterberg, Netherlands) for 30 min according to the manufacturer’s recommendations.

### Flexural strength tests

Three-point bending tests were applied to measure flexural strength using a universal testing instrument (EZ-LX, Shimadzu, Kyoto, Japan). For each group, calipers were used to check the dimensions of each specimen. The maximum loading force (*F*) at the time of fracture was recorded in newtons after specimens were placed on the holder (Fig. 5B,C). The flexural strength ($\sigma$) was calculated in megapascals using the following equation (Eq. 1):

1 $$\sigma = \frac{3FL}{{2wh^{2} }}$$

where *L* is the span length between the supports in millimeters, and *w* and *h* are the width and height of the specimen in millimeters, respectively.

### Vickers hardness

Specimens were manufactured and then tested immediately using a Vickers hardness tester (MMT-X7, Matsuzawa, Kyoto, Japan). A loading force of 0.49 N was applied for 10 s to the surface opposite the first printed side of the specimen. Each specimen was measured three times, from which the mean value was calculated in gigapascals.

### Scanning electron microscopy

The scanning electronic microscopy (SEM; S-3000 N, Hitachi, Krefeld, Germany) was utilized to capture the features of the sample surfaces. The specimens were mounted on the holder after coating with platinum. All images of each group were acquired at 1500× magnifications.

### Degree of conversion

The degree of conversion (DC) was measured for each specimen and unpolymerized resin using a Fourier-transform infrared spectrometer (FT-IR; Nicolet iS10, Thermo Fisher Scientific, Waltham, MA, USA). The surface opposite the first printed side of each specimen was directly measured three times. After baseline correction, the DC was calculated as follows (Eq. 2):

2 $$DC\left( \% \right) = \frac{{\left( {\frac{{ {\text{C=C}}_{monomer} }}{{{\text{C=O}}_{monomer} }}} \right) - \left( {\frac{{ {\text{C=C}}_{polymer} }}{{{\text{C=O}}_{polymer} }}} \right)}}{{\left( {\frac{{{\text{C=C}}_{monomer} }}{{{\text{C=O}}_{monomer} }}} \right)}} \times 100\%$$

the absorption bands of ${\text{C=C}} _{monomer}$ and ${\text{C=C}}_{ polymer}$ peak at 810 cm^–1^ before and after exposure to the UV light, respectively, and ${\text{C=O}} _{monomer}$ and ${\text{C=O}}_{{{ }polymer}}$ are the absorbances at 1720–1730 cm^–1^ before and after exposure to the UV light.

### Biocompatibility assay

1. Cell culture
  Primary human gingival fibroblasts (HGFs; PCS-201-018, ATCC, Manassas, VA, USA) were cultured in a dish with Dulbecco’s modified Eagle’s medium (DMEM, WELGENE, Daegu, Korea), supplemented with penicillin/streptomycin (100× , WELGENE), minimum essential medium nonessential amino acid solution (100× , WELGENE), and 10% fetal bovine serum (Thermo Scientific, Waltham, MA, USA). Cells were cultured under an atmosphere at 37 °C, 5% CO_2_, and 100% relative humidity until they achieved 85–90% confluence. Cells were then seeded onto 96-well plates at a density of 8 $\times$ 10^3^ cells/well after they were treated by EDTA (trypsin-ethylenediaminetertraacetic acid) solution (WELGENE). After 24 h of incubation at 37 °C and 5% CO_2_, the original medium was replaced by the resin extract at 100 μL/well. Cells were allowed to grow at 37 °C and 5% CO_2_ for a further 24 h before the cell viability assays were performed.
2. Preparation of extraction
  The specimen extracts were made after sterilization with ethylene oxide gas. The extraction ratio was 1.25 cm^2^/mL following the ISO 10993-12 standard. The specimens were then immersed in a tube with DMEM solution and stored at 37 °C for 24 h. The extracts were used within 24 h of preparation.
3. Cell viability assay
  CELLOMAX™ viability kits (Precaregene, Anyang, Korea) were prepared based on the WST-8 tetrazolium salt [2-(2-methoxy-4-nitrophenyl)-3-(4-nitrophenyl)-5-(2,4-disulfophenyl)-2H-tetrazolium and monosodium salt; Precaregene, Hanam, Kyungido, Korea]. After the cells had been cultured for 24 h, 10 μL of CELLOMAX™ solution was added to the 96-well plate and the entire plate was incubated for 90 min at 37 °C according to the manufacturer’s instructions. A microplate reader (VERSA max, Molecular Devices, Sunnyvale, CA, USA) was used to record the optical density (OD) at a wavelength of 450 nm. Cell viability was calculated as follows (Eq. 3):
  3 $$Cell \,Viability \left( \% \right) = \left( {\frac{{ OD_{test\, sample} - OD_{blank} }}{{OD_{control} - OD_{blank} }}} \right) \times 100\%$$
4. Cell morphological observations
  An inverted microscope (Eclipse TS 100, Nikon, Tokyo, Japan) was used to visualize the morphology and density of HGFs cultured in the extracts of all groups of specimens for 24 h, and the changes were compared.

### Gas chromatography

Gas chromatography/mass spectrometry (GC–MS) was used to detect the eluted substances after the different postwashing processes. Samples were immersed in 1 mL of acetone in tightly sealed brown glass vials and incubated at 37 °C for 24 h after postprocessing. Elutes were then measured by GC–MS (7890B/5977 A series gas chromatograph/mass-selective detection system, Agilent, Santa Clara, CA, USA), with the relevant compounds identified by comparing their mass spectra and retention times with the corresponding reference standards. A calibration was performed for each reference-standard monomer. The quantity of an analyte was calculated by correlating its characteristic mass peak area with the corresponding precompiled calibration curve.

### Statistical analyses

Standard statistical software (SPSS version 26.0, IBM, Armonk, NY, USA) was used for statistical analyses. The presence of normality was checked using the Shapiro–Wilk test. Two-way ANOVA with Bonferroni post-hoc tests was applied to evaluate how the different washing agents and washing times influenced the flexural strength, Vickers hardness, and DC results. Two-way ANOVA followed by Turkey post-hoc tests was used to evaluate the results from the cell viability assay. The significant cutoff was set at 0.05.

## Conclusions

This study investigated the effects of washing agents and washing times on a water-washable 3D printing C&B resin, and four main conclusions can be drawn. First, the mechanical properties of the resin weaken with increasing washing time. Water and detergent washing agents are better than alcohol for preserving the mechanical properties during an extended washing process. Second, SEM observed that the resin washed with water and detergent had a smoother surface than that washed with alcohol. Second, the DC increases with the washing time when using water and detergent dilutions, whereas it decreases when alcohol is used. Third, increasing the washing time significantly improves cell viability for a resin washed with water. After 20 min of washing, there was almost no significant difference in the cell viability of the resins washed using different washing agents. Fourth, resin polymer releases different amounts of monomers for different washing agents and times, with no simple linear relationship between them.

Recent articles observed the impact of ethanol treatment on medical resin properties. Similar results can be obtained, ethanol will cause a decrease in the mechanical properties of the treated resin^16,58^, and reduce cytotoxicity^59^. The conclusions of this study are in line with the previous findings. The research outcomes have drawn the attention of medical researchers toward the selection of appropriate agents for treating medical resins. This also led to further exploration of various postwashing processes for optimization in clinical environments. Ultimately, these efforts aim to extend the long-term service life and effectiveness of medical resin materials.

The water-washable 3D printing temporary crown and bridge resin can be washed with water without compromising its mechanical properties and biocompatibility, making water a dependable agent option for washing the 3D printing water-washable intraoral resin. This advancement encourages the development and clinical application of other types of water-washable resins in dentistry. Furthermore, the washing agents and washing time were evaluated to enhance subsequent clinical standardized washing guidelines.

## Data Availability

The data that support the figures and tables within this paper are presented in the main text. All other additional data are available from the corresponding author upon request.
